# Morphological rectal alterations following STARR performed for obstructed defecation syndrome

**DOI:** 10.1186/1471-2318-11-S1-A51

**Published:** 2011-08-24

**Authors:** A Reginelli, Y Mandato, A Russo, F Iacobellis, S Cappabianca, L Brunese, R Grassi

**Affiliations:** 1 Section of Radiology, Department ‘‘Magrassi-Lanzara,’’ Second University of Naples, Naples, Italy; 2 Department of Radiology, Health Science, University of Molise, Campobasso, Italy

## Background

Anorectal functional disorders are common disease involving about a quarter of population. Most patients concerned are females complaining of one or more symptoms, with a negative impact on quality of life, and high social costs. Traditional surgical treatments are frequently unsatisfactory, indeed some surgeons opt for conservative therapy consisting in the pelvic floor rehabilitation. A new surgical technique, the STARR (stapled trans-anal rectal resection) seems to show encouraging results. STARR aims to improve obstructed defecation syndrome (ODS) symptoms by the correction of the associated rectal anatomical alterations, such as rectocele and/or rectal intussusception. Contraindications to STARR are represented by stenosis, perianal sepsis, malignancy, enteroceles, sigmoidocele.

## Patients and methods

Thirty-five patients were investigated by dynamic defecography before and after STARR. Preoperative imaging findings were: rectocele, recto-rectal intussusceptions, internal mucosal rectal prolapse, full-thickness rectal prolapse, descending perineum and paradoxical pubo-rectalis syndrome. Four and six months after surgery, defecographic follow-up was carried out.

## Results

In only one case, the rectal intussusception was not corrected by surgical treatment; in two patients, partial reduction of rectocele was found. Two patients, in which the STARR corrected functional and anatomical defects, showed a iatrogenic rectal diverticulum anterior and posterior respectively. In thirty patients, an “hourglass” stricture was found and the rectal ampulla was reduced in size and length.

## Conclusions

The STARR represents an effective surgical treatment in patients with ODS symptoms. Dynamic defecography is a useful method to define patients who require surgical treatment and to investigate the causes of surgical failure or relapses.

